# New Trends in Anterior Segment Diseases of the Eye

**DOI:** 10.1155/2014/393040

**Published:** 2014-08-05

**Authors:** Francisco Javier Romero, Bjørn Nicolaissen, Cristina Peris-Martinez

**Affiliations:** ^1^Facultad de Medicina y Odontologia, Universidad Católica de Valencia “San Vicente Mártir”, C/Quevedo 2, 46001 Valencia, Spain; ^2^Center for Eye Research, Department of Ophthalmology, Oslo University Hospital and University of Oslo, Pb 4956 Nydalen, 0424 Oslo, Norway; ^3^Fundacion Oftalmológica del Mediterraneo, Bifurcación Pio Baroja/General Aviles, 46015 Valencia, Spain

Disorders of the anterior segment of the eye are leading causes of ocular morbidity. Such conditions include dry eye conditions, infections, traumas of various types, inflammatory reactions, hereditary disorders, and cataract. For a number of these patients, the rule is a continuous progression and aggravation of symptoms. The end stage is varying degrees of visual loss with or without pain.

Despite continuous advances in ophthalmology, a number of these patients represent a challenge even in highly specialized clinics in western countries. On a worldwide basis, such conditions represent a significant public health problem. Cataract and loss of corneal transparency are still two of the most common causes of blindness worldwide [1]. While cataract is a disorder of the adult and aged population, blindness due to corneal opacities is observed in all age groups. In children, loss of corneal transparency represents the third most common cause of blindness.

During the past two decades, translational research has increased our ability to understand the pathogenesis of, and also to treat, selected disorders of the ocular surface and cornea. Although still in their shaping and improved by continuous translational and clinical research, procedures for ex vivo production of corneal and conjunctival epithelial tissue allow treatment of patients with previously untreatable corneal disorders [2, 3]. Refinement of corneal transplant procedures permits targeted intervention and replacement of opaque and nonfunctioning tissues with lamellar donor tissue [4, 5]. By such techniques, the volume of foreign tissue with a potential for stimulation of immunoreactions is reduced as is the trauma induced by the surgical intervention. However, there is a pronounced lack of donor corneas. In western countries, one main indication for corneal transplantation is loss of endothelial function. Ongoing research within the field of tissue engineering will provide procedures for production of transplantable layers of corneal endothelium and thereby add a new and significant tool to our treatment options [6]. Procedures for cataract surgery are continuously being advanced, accompanied by a decline in the rate of complications such as astigmatism and corneal endothelial loss with subsequent corneal hydration [7, 8]. Due to the complexity of challenges within these areas, further progress relies to a significant extent on interaction between clinical and basic research environments. In translational research projects, extraction of information is facilitated by cooperation between clinical and basic research environments [9, 10]. The intent is to warrant that advances in basic and clinical knowledge may serve a purpose: a better understanding of the disease pathophysiology to ensure a better disease prevention, new diagnostic procedures, and novel types of treatment including drugs, whose final end point may be preclinical or clinical testing.

*Francisco Javier Romero* *Francisco Javier Romero* *Bjørn Nicolaissen* *Bjørn Nicolaissen* *Cristina Peris-Martinez* *Cristina Peris-Martinez*
